# Machine Learning approaches for the detection of disease-causing variants in whole-genome data need to address the expression of functional genes

**DOI:** 10.1371/journal.pone.0355557

**Published:** 2026-08-28

**Authors:** Camilla Mapstone, Julia Handl, David Talavera

**Affiliations:** 1 Division of Cardiovascular Sciences, School of Medical Sciences, Faculty of Biology, Medicine and Health, The University of Manchester, Manchester, United Kingdom; 2 Alliance Manchester Business School, The University of Manchester, Manchester, United Kingdom; Calico: Calico Life Sciences LLC, UNITED STATES OF AMERICA

## Abstract

Gene-dosage combinations have been recognised as leading factors of disease. Given that those combinations may include dozens of genes, it is hypothesised that machine learning (ML) approaches may be useful in the classification of cases and controls and the identification of causative genes. We aimed to assess the validity of this hypothesis. Here, we have constructed a benchmark that includes real data (with ground truth knowledge) and synthetic data with known generating mechanisms and various dataset sizes and levels of noise. We trained standard statistical learning/ ML models on these datasets to classify disease phenotype. We present an analysis of how model performance varies across different synthetic genetic scenarios, and how it is impacted by dataset size. The logistic regression model was found to be the most reliable at causative gene identification across the synthetic datasets, despite not always performing the best in terms of classification performance and, in some cases, having a relatively low ROC AUC score. When our training attempts on the UK Biobank datasets failed, we performed an analysis into model performance vs dataset richness. Our results show that it is necessary to take into account the expression of functional genes in order to successfully predict disease.

## Introduction

Although some seminal work did not find common Copy Number Variants (CNVs) to be a major contributor to the heritability of common diseases in humans [1], it is becoming clear that rare CNVs have an effect in multiple anthropometric traits and diseases such as neurodevelopmental and neuropsychiatric disorders, obesity and cardiovascular malformations [2–14]. It is assumed that some CNVs are likely to exert their effect through gene dosage imbalances [14–18]. Protein complexes, regulatory networks or signalling pathways have correlated gene expression [19,20], hence alterations in gene dosage might affect their transcript or protein stoichiometry [21]. The Gene Dosage Hypothesis has been criticized as too simplistic to be applied to all gene-copy variants [22,23]; i.e., overall correlation between gene dosage and amount of protein is weak, because gene expression is regulated at multiple levels –transcription, translation and proteolysis. Nonetheless, there is growing evidence for a set of genes sensitive to dosage balances depending on their position within a network of protein interactions [24,25], their involvement in specific pathways [11,23,26] and/or their tissue-specific expression [26,27].

Some studies have been able to identify the specific genes whose deletion or duplication is associated with diseases [7,11,28–32]. Nonetheless, more often, CNV analysis can only uncover associations with multi-genic regions [4,6,10–13,33]. A striking example of this is the lack of conclusive results in the identification of genes associated with Atrioventricular Septal Defect within chromosome 21 [34] even though it is well known that this cardiac malformation is highly prevalent in individuals with Down Syndrome [35]. There are several reasons for this slow advance in narrowing CNVs to specific genes: 1) patients with clinically-similar phenotypes may have non-overlapping CNVs, sometimes even in different chromosomes [36,37]; 2) the same CNVs are associated with disparate traits and diseases [2,4–6,10–13]; and, 3) CNVs associated with diseases are also found in healthy individuals. To exemplify these points, the 15q11.2 BP1-BP2 deletion has been associated with both neuropsychiatric disorders and congenital heart disease [4,13,38–43] though it is not the only CNV associated with these diseases [3,4,38,41,42]; its prevalence is 0.57%−0.68% in patients with neurodevelopmental, neuropsychiatric or congenital diseases, and 0.25%−0.38% in healthy cohorts [13,44].

The multigenic nature of many traits and diseases [45–51] and the well-documented contribution of epistatic relationships to phenotypes [52–55] can explain some of the difficulties in ascertaining the genetic causes of diseases. According to those models [17,18,49,50], many diseases are caused by various specific combinations of variants. The involvement of many genes in each disease helps to explain the pleiotropy associated with many CNVs [49–51]. These hypotheses are supported by evidence showing that not all gene deletions are equally relevant as causes of disease [11,16,23–25] and the effects of the deletions might be tissue-specific [11,16,27].

This combinatorial aspect of variants suggests that Machine Learning (ML) approaches should be poised to thrive for the prediction of outcomes or the identification of causative features. While ML algorithms may be able to simultaneously discriminate between cases and controls (a classification problem) and identify the discriminant gene-dosages (a feature selection problem), these are different problems from a clinical/biomedical point of view. For example, if we wanted to identify and treat people at risk of having a heart attack based on their genome-wide gene-dosages, we would need an excellent classification performance. Conversely, if a baby was born with a cardiac defect, we would already have a clinical diagnosis; so, the classification achieved through the ML approaches would be irrelevant. Nonetheless, we would still be interested in understanding why that defect was developed. Although there have been attempts at predicting disease risks based on combinations of gene dosages [56], most of the applications of ML algorithms to the field of gene-dosage research have aimed to predict the pathogenicity of individual gene dosage alterations [14,26,57,58].

In this work we aimed to assess the performance of various standard ML algorithms in the classification of disease phenotype (case or control) caused by different genomic scenarios. Moreover, we were interested in understanding the level of classification performance necessary to obtain reliable estimates of feature importance. When the good performance observed in synthetic data was not reproduced in real biological data, we investigated the possible reasons for this poor performance and strategies to improve it.

## Results

### Modelling on synthetic datasets

**Fig 1:**
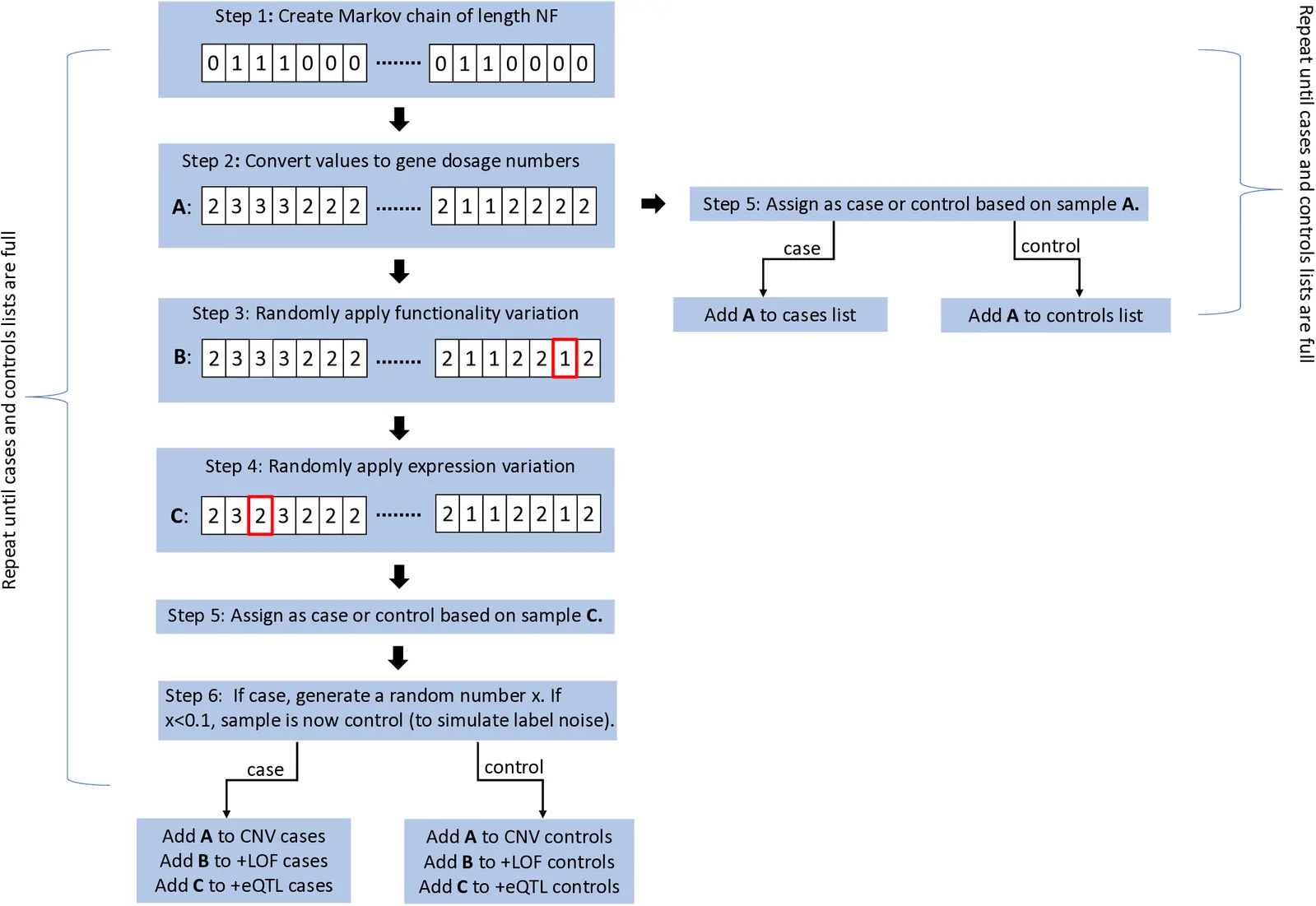
Methodology for creating synthetic datasets. Both methodologies used in these studies are shown, the main branch illustrates creation of datasets for the investigation into the effect of dataset richness (Fig 4), and the right branch illustration the creation of datasets for the investigation into the effect of dataset size (Fig 2).

To test the capability of various models in predicting disease and detecting causative genes we created synthetic datasets (see Fig 1) representing gene dosages for a number of cases and controls. We defined 5 types of disease scenarios –labelled A to E- based on the number of causative genes and their additive or epistatic relationships. Disease scenarios are summarized in Table 1: some of the scenarios treated deletions and duplications equally, while others treated them differently; in some scenarios, just the number of gene dosage alterations was important, while in others, changes had to occur in specific pairs of genes. The dataset consisted of a number of simulated samples (range used was 50–10,000), with each sample having 1,000 features. Each feature represents a gene; a minority of features are causative features (CF) –equivalent to causative genes- while the rest contribute noise. For each disease scenario, a set number of features were designated as CFs and were spaced evenly throughout the vector of features to simulate the existence of causative genes in different chromosomes. The feature values were set randomly as 1, 2, 3, or 4, representing the copy number of that gene (we only considered full-length gene copies hence the integer values). Values were drawn from an empirical distribution reflecting frequencies in real biological data (see Methods for details). We did not allow values of 0 or values greater than 4 as these were not observed in the real biological data, so we assume that only copy numbers of 1–4 are possible. The majority of features have a value of 2, the normal number of copies of a gene (i.e., one paternal copy of the gene and one maternal copy of the gene); features with values other than 2 are said to have a copy number variation (CNV). A sample is defined as a case or control depending on a specific condition defined by the disease scenario regarding the values of the CFs. No other factor (e.g., environment, age, sex or ancestry) contributes to the definition.

**Table 1:** Definition of genetic scenarios.

Scenario	Definition	Number of different causative combinations^1^
A	At least one CF out of a set has a CNV	3×n
B	Both genes within at least one pair of CFs have a CNV.	$\text{3}\times \frac{n}{\text{2}}$
C	At least one CF from each of two sets of CFs needs to have CNVs	$\text{3}\times {\left(\frac{n}{\text{2}}\right)}^{\text{2}}$
D	At least one CF out of a set has a CNV and the type of CNV is specified (duplication for half the CFs, deletion for other half of CFs)	$\text{2}\times \frac{n}{\text{2}}+\frac{n}{\text{2}}$
E	Both genes within at least one pair of CFs have a CNV. Alternatively, at least 3 CFs have a CNV	$\text{3}\times \left(\frac{n}{\text{2}}+\frac{n!}{\text{3}!(n-\text{3})!}\right)$

^1^*n* equals the number of CF. Each CF can have 1 deletion value and 2 duplication values.

Scenarios A and D represent monogenic diseases with several causative genes; i.e., a gene-dosage alteration in any of the causative genes will result in a disease phenotype. Scenarios B and C represent digenic diseases in which the disease is caused by concurrent gene-dosage alterations in particular combinations of causative genes [59]. These are clearly epistatic scenarios [60]; i.e., individual alterations are harmless, but their combination is deleterious. The main difference between the scenarios is that each gene is involved in a single causative combination in scenario B, whereas genes can be involved in multiple causative pairings in scenario C. Scenario E is a combination of Scenario B plus a trigenic disease. It could be seen as an oligogenic disease with epistasis; i.e., three variants are generally necessary, but two variants can be enough to cause the disease if they occur in specific pairs of genes.

The number of CF (*n*) used for each scenario was determined by choosing the amount that would yield a population ratio of cases to controls (pRCC) of approximately 0.1, based on a CNV frequency value of 0.05 for all features. As a reference, pRCC ≈ 10% is similar to the prevalence for diabetes mellitus [61] and chronic kidney disease [62] in middle-age and old-age populations, a bit lower than the prevalence of hypertension [63] and a bit above the prevalence of coronary artery disease [64], and chronic obstructive pulmonary disease [65]. The value of 5% of CNVs is a high overestimate to allow for enough features (both causative and non-causative) to contain CNVs. We used the UK Biobank [66] to estimate a CNV frequency equal to 0.0003. This means that on average 6 protein-coding genes have a CNV in each genome. As we were only using 1,000 features, our mean estimate would be of 0.3 CNVs per sample. Even if we aimed for 6 CNVs per sample (as estimated in the real data), our dataset would be much simpler than real genomes; since we were not considering other types of variation, there would be very few noise features with a CNV in each sample.

For each scenario, datasets were created with both this initial *n* value, and also a value of *n* increased or decreased by a factor of 10, in order to explore how the modelling outcomes for each scenario varied depending on *n*; i.e., we simulated equivalent monogenic, digenic or trigenic scenarios with different population class imbalances. Through testing various *n* values via the random creation of 1,000,000 CF sets for each value, we determined that values of 2, 100, 12, 4, and 20 yield population RCCs of around 0.1 (see Table 2) for scenarios A-E respectively. Scenarios are referred to here as the scenario group letter + *n* (e.g., scenario A with 2 CFs is A2), we created datasets for scenarios A2, A20, B10, B100, C12, C120, D4, D40, E20, and E200 in this study. Given that CNVs can include deletions (i.e., gene-dosages equal to 1) and duplications (gene-dosages equal to either 3 or 4), the number of possible causative combinations ranges from 6 in A2 and D4 to 3,940,500 in E200. This latter scenario includes 300 pairs and the rest are triplet combinations.

**Table 2:** Population RCC for each scenario, based on 1,000,000 random samples.

Scenario	pRCC
A2	0.098
B100	0.119
C12	0.071
D4	0.096
E20	0.084

The synthetic datasets created all had a controlled RCC of 0.2 (note that this value is greater than the pRCC as in most studies the number of cases in the datasets exceeds the prevalence in the population). Each dataset was created by randomly producing samples, determining whether the sample was a case or a control based on the CF values, and then adding the sample to either the set of cases or set of controls. This process continued until each class was full. As all samples were assigned their correct label, these experiments did not include any label noise; i.e., diseases had complete penetrance and neither misdiagnosis nor late-age onset were considered. To create a sample, we first created a Markov chain that produces binary data with a steady state probability equal to the CNV frequency, then converted the numbers in this chain from 0 to 2 to represent the normal state and 1–1, 3, or 4 to represent the CNV states (see Methods). Using a Markov chain ensures that CNVs are not randomly spread through the whole synthetic genome, but they can involve multiple features; i.e., in the case samples, features adjacent to CFs may also have a higher chance of having a CNV. Full details of this process are detailed in the Methods section. For each scenario, we created datasets with varying number of samples (NS) to see the effect of sample size on model performance, as we expect overfitting to become an issue as sample size is reduced. Multiple datasets were created for each NS value to allow averages and standard error to be calculated for model performance metrics. A higher number of datasets were created for smaller NS values to account for the higher uncertainty levels associated with the correspondingly smaller test sets (each dataset was randomly split into training and test sets with a 80:20 split). The ML algorithms tested on each dataset were Logistic regression (LR) with and without L1 regularization, decision tree (DT), random forest (RF), and a neural network (NN) with and without L1 regularization. Class imbalance was handled using the class_weight parameter for all models. Categorical cross entropy was used for the NN and the Scikit-Learn default was used to optimise all other models. It is worth noticing that none of these algorithms use spatial information. Thus, even if CNVs tend to be grouped in short runs within each sample, this information is not used.

**Fig 2:**
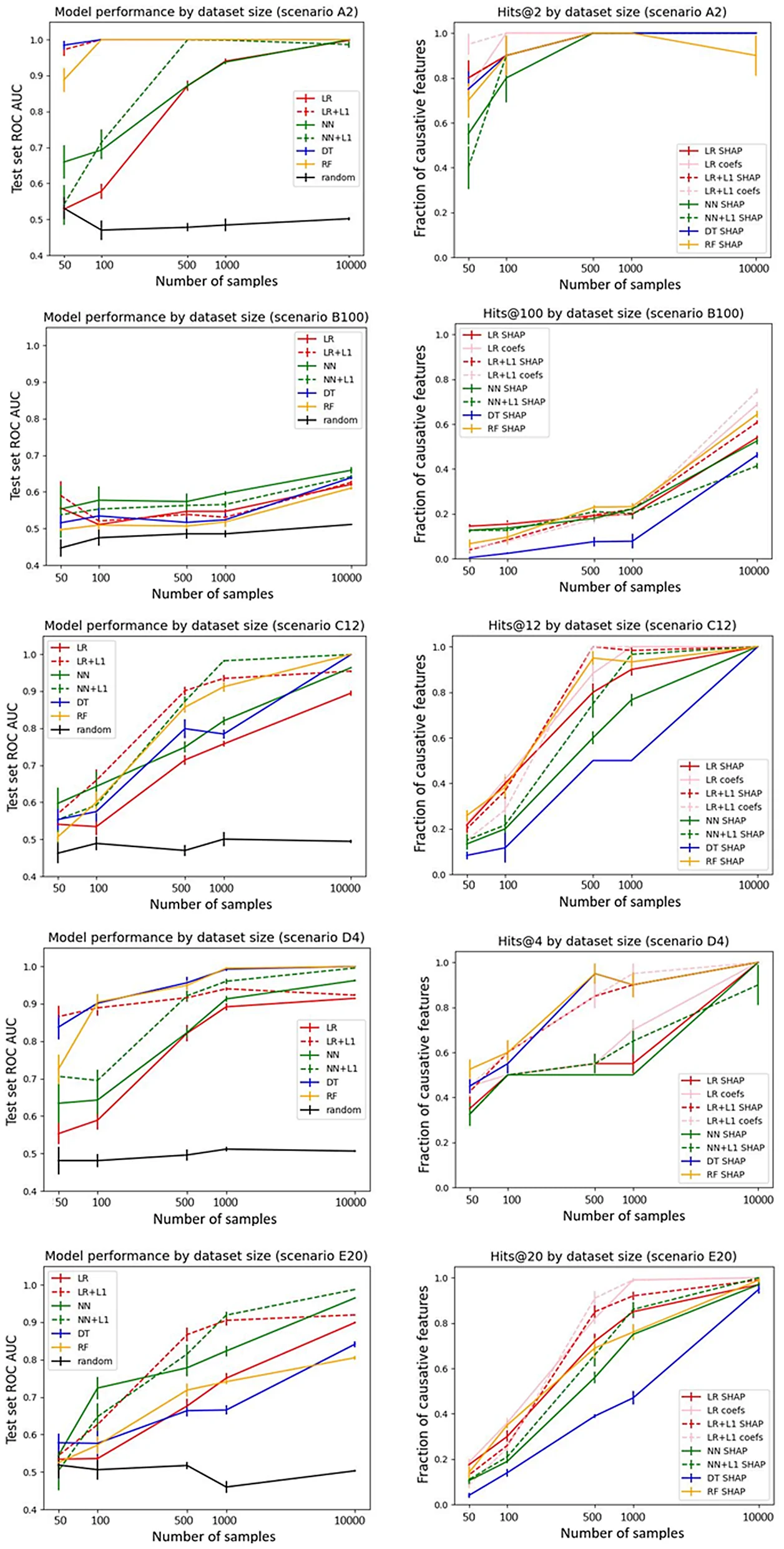
Model performance metrics by number of samples for scenarios A2, B100, C12, D4, and E20. For each scenario, the test set ROC AUC and Hits@K are shown for dataset sizes from 50−10,000.

For each dataset we calculated four different metrics for each model. The first was Area Under the Receiver Operating Characteristic Curve (ROC AUC) of the test set; for this the dataset was split into a training and test set, with an 80:20 split, and all models were trained on the training set and evaluated on the test set (see S1 Fig). This gives us a general idea of how well the model can predict the simulated disease and is our only metric focusing on classification performance. 5-fold cross validation on the training data was used for hyper-parameter tuning when training the LR, DT, and RF models, with the model then re-trained using the whole training set and the best found hyperparameters. All the following metrics measure how reliable the model is at identifying causative features. The second metric was Hits@K, which tells us what proportion of the top ranked features are actually CFs, so indicates how useful the model is at identifying CFs. For this we trained the model on the whole dataset (again, using 5-fold cross validation and re-training with the best found hyperparameters) and calculated SHapley Additive exPlanation (SHAP) values, and also extracted model coefficients for the LR models. We used the whole dataset to calculate the SHAP values as more training data should increase overall model performance, and in practice a test set would not be necessary if a dataset were used for this purpose of causative gene identification as the aim would not be to predict disease. To calculate the SHAP values we used either all samples or 100 randomly selected samples (whichever value was lower) as calculating SHAP values was computationally expensive so not feasible for all samples in large datasets. To calculate Hits@K we took the top *K* features as determined by the SHAP values/coefficients and worked out how many were CFs, with *K* equal to *n*. If the SHAP value or coefficient of a feature in the top *K* features was 0 (a frequent occurrence), that feature was automatically not counted as a hit. The final two metrics are number of CFs found and last rank, defined as the number of CFs with non-zero coefficients/ SHAP values and the rank of the lowest ranked non-zero CF, respectively. This gives us an idea of how many CFs could be identified, and the prevalence of false positives. For these the SHAP values and LR model coefficients were also used. The values of these metrics for each model and each scenario are shown in Figs 2 and S2–S4.

The results show that the performance of the models relative to each other depends on the disease scenario, and as expected all models generally perform better as NS increases and struggle when increasing *n* (Figs 2 and S2). Nonetheless, it can be seen that, for the vast majority of scenarios, it is possible for at least some of the models to obtain a high performance if there is enough data. For the A scenarios the LR obtains a ROC AUC equally as high as other models, but as expected it tends to perform less well than the NN and sometimes also the DT and RF for the other scenarios when there is non-linearity present. However, the LR coefficients often achieve the highest Hits@K score and can even achieve a high Hits@K score when the LR model did not obtain a particularly high test set ROC AUC score (Figs 2 and S2 and S1 Table). Therefore, the LR model may be less useful when a classification is desired (e.g., as a risk predictor), however may still be useful for diseases where classification is irrelevant but causative genes are unknown (e.g., as mechanistic knowledge generator). Finally, S3 and S4 Figs show that, as the number of samples increase, the algorithms can more easily identify the true CFs and separate them from the non-CFs. DT seems to be the most struggling algorithm when examining those metrics.

### Testing model on UK Biobank data

We next wanted to see how well the models would work on a real dataset. For this we attempted to train models to diagnose coronary arterial disease (CAD) and bicuspid aortic valve (BAV) using samples from the UK Biobank. The former disease is primarily a risk prediction application, while the latter one focuses on the identification of causative features as BAV is a congenital disease. We attempted to train models to classify CAD vs control, BAV vs control, and also BAV vs age-related aortic valve disease – a cohort of samples over 65 years old with aortic valve disease. A summary of each cohort along with sample numbers is provided in Table 3. Due to the large dataset sizes, we randomly sampled from the CAD and control cohorts, selecting 4000 samples from each. Although larger samples lead to better performances (Figs 2 and S2–S4), the computational cost of training models increase exponentially with the size of the dataset. 8000 samples is close to our larger synthetic dataset; however, the number of features in the real-world data is much greater.

**Table 3:** Summary of UK Biobank cohorts.

Cohort	Description	Size
CAD	samples with coronary arterial disease.	22138
BAV	Samples with bicuspid aortic valve that include those with specifically ‘congenital’ diagnostic codes as well as those that we infer from them having an aortic valve disease diagnosis <=65 years old.	1163
65AV	samples with aortic valve disease >65yrs.	2810
ctrls	A cohort that can act as controls for all of the above cohorts. The samples do not contain any of the diagnostic codes used to classify BAV, av_over65 or CAD. They also do not contain any individuals with syndromic conditions that might make them unsuitable for controls.	440906

We found that it was not possible to train any of the models we used previously on the synthetic datasets to perform above chance, defined by a ROC AUC greater than 0.5 for the test set. We carried out training attempts with and without data standardization, with and without feature value adjustment (2s replaced with −1 to create a linear separation between ‘normal’ and ‘abnormal’ features values), and with various hyperparameter values for the NN. Although some approaches increased the training set performance, none were able to generalize to the test set therefore we still did not find any modelling framework that performed better than chance. Given that all the ML models performed well in the synthetic data provided that there were enough samples, we speculate that the reason for the poor performance when using real-world data is not due to limitations of the ML models but to the complexity of biological data, which warrants further exploration.

**Fig 3:**
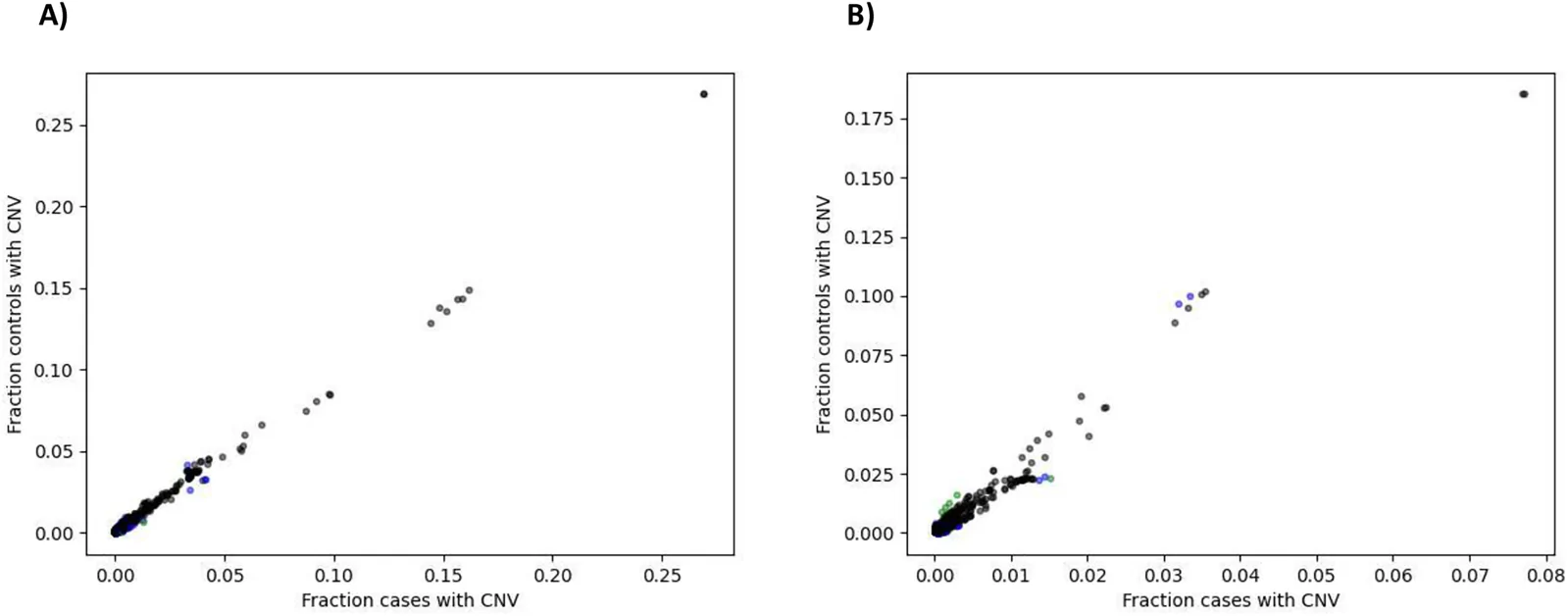
Distribution of CNVs in real biological datasets. A) Fraction of CAD samples (cases) vs fraction of control samples that had a CNV for each gene. B) Fraction of BAV samples (cases) vs fraction of av_over65 samples (controls) that had a CNV for each gene. Genes for which the fraction differences appeared to be statistically significant under individual testing were color coded blue for pvalue<0.05 or green for pvalue<0.01. The reported p-values are not corrected for multiple testing. None of those differences remained statistically significant after multiple testing correction: i.e., none of them reached genome-wide significance.

To investigate why our models failed to perform, we examined how often CNVs were found in each gene for the different cohorts. We then compared these frequencies for CAD vs ctrls and BAV vs 65AV (Fig 3). The results show that most points lie close to the diagonal. When assessing each gene individually in terms of enrichment or depletion in the number of CNVs, we found that some differences appeared statistically significant (S2–S3 Tables in S1 File); however, none of those remained significant after correcting for multiple testing (S2 Table). Therefore, no single gene may be presumed to be the main contributor to these diseases when using a genome-wide approach. Diseases are likely to be caused by particular combinations of gene dosages. Alternatively, these diseases could include a high percentage of late-onset cases or missed diagnoses.

Out of curiosity, we decided to investigate if the features with the greatest contribution in the LR model were somehow related to the diseases that we were studying (S5–S10 Tables in S2 File). The rationale for this analysis was that LR was able to identify many CFs in the synthetic datasets even when the classification performance was not great (Figs 2 and S2). With real data we do not know how many *bona fide* causative features there are; so, we analysed the top 100 features in two experiments (BAV-65AV and CAD-ctrls). 32 features were top-contributors in both experiments supporting the hypothesis that the intrinsic structure of the data makes the classification task really hard for the ML algorithms. As expected with such poor performances, most of those features were totally unrelated to aortic valve diseases or coronary arterial disease. Nonetheless, there were a few surprising findings pointing to a link between some of the features and the diseases: 1) DAVID [67] identified a group of genes (KCNH2, AGBL4, ERBB4) linked to atrial fibrillation, which has been associated with aortic valve disease [68–71]; 2) g:Profiler [72] identified a group of genes (CFHR1, FCGR2B, HLA-DRB1, CFHR3) influencing the concentration of complement C3, which has been observed to increase in case of coronary artery disease [73]; and, 3) DAVID identified a group of genes (MSRA, CTNNA3, AHR, CYP2C19, SGCZ, HLA-DRB1, CADM2, CYP2E1) linked to coronary artery disease and myocardial infarction. Although it is probable that these are just serendipitous findings, we do believe that the finding that simple ML algorithms –which are less sensitive to overfitting- may be able to identify some true causative features even when their classification performance is poor, is an exciting perspective which warrants further research in the future.

### Investigating the effect of dataset richness on model performance

To further investigate why the models failed to perform on the UK Biobank data, we next investigated the performance on simulated datasets with incomplete information and label noise (see Fig 1). In reality, it is not just CNVs that are the genetic cause of disease. Even if there are the correct number of physical copies of a gene, that gene can be non-functional or over/under-expressed.

To carry out this analysis, a dataset of CNVs was first created using Markov chains as before, and then elements in the chain were randomly picked and their dosage increased or decreased by one to simulate the presence of variants affecting the expression of functional copies (see Methods). Loss-of-function variants (LOF) were always represented as a subtraction, while variants resulting in alterations of normal gene expression levels (eQTL) could either increase or decrease the gene dosage (i.e., they were represented as an addition or a subtraction, respectively). We followed the same steps as before to create datasets of a controlled RCC, however each time a sample was generated we created three versions of the sample with increasing richness of information (each subsequent version of the sample contained additional layers of information). The first version of the sample was the original Markov chain representing just copy numbers, this was added to the ‘CNV’ dataset. The second version was the Markov chain with gene-functionality status information added, this was added to the ‘+LOF’ dataset. The final version of the sample, which also contained information on variants affecting gene expression, was added to ‘+LOF+eQTL’ dataset. The case and control labels were assigned to the samples in the + LOF + eQTL dataset based on the number of expressed functional copies; i.e., the correct label is a function of the three mechanisms. After adding the correct labels to the + LOF + eQTL dataset, some label noise was added to the dataset by switching some case labels to control. The labels in the + LOF + eQTL dataset were used across all three datasets; i.e., labels in the + LOF + eQTL dataset were copied to the corresponding samples in the CNV and +LOF datasets. This data generation protocol means that many of the samples in the CNV and +LOF datasets did not contain all the layers of information necessary for the label to be inferred; i.e., they contained incomplete information.

The relative frequencies of the different types of variants needed to represent how frequent these all are in comparison to each other in reality. Using real datasets [66,74], we estimated a frequency of 0.0003, 0.0008, 0.0002, and 0.0008 for CNVs, LOF variants, variants increasing the expression of the gene copy, and variants inhibiting the expression of the gene copy respectively. The CNV frequency was calculated from the overall frequency of all CNVs in the control group, LOF variants were estimated from previously characterised exome data [75], and the expression variants were chosen so that they were in the same order of magnitude as the other variants and in overall agreement with some previous estimates [76]. As before, we used 1000 features to reduce computational costs rather than the approximately 20,000 coding genes that might be included in real datasets. Given that the expected number of variants per sample is quite small, we multiplied all frequencies by 20 so that the spread of variants in CF and non-CF can be more representative of real datasets; i.e., otherwise, variants may rarely be found anywhere other than the CF of case samples. The overall frequency of gene dosage variation is then 1 – P(no gene dosage variation) = 1 – P(no CNV)*P(no LOF)*P(no expression variation) = 1- (1-0.006)*(1-0.016)*(1-(0.004 + 0.016)) =0.0415. This is close to the value of 0.05 we had been using before, therefore we kept the *n* values we had been using previously. Given that our synthetic dataset had an equivalent number of variants as real biological datasets, our limit to 1,000 features can be seen as if we clustered all the variants into those features and made the remaining 19,000 features invariable. Although this is not biologically realistic –the proportion of invariant genes is much lower than 95% (see details on dimensionality reduction in the Methods section)-, it is necessary in order to have a tractable problem with enough variants. The simulated datasets were intended to represent the CAD vs control scenario, so we wanted to use the same ratio of number of features to number of samples. Therefore, we divided the number of samples by 20 to get an NS value of 400 and kept the sampled RCC at 0.5. In addition, we added label noise by adding some cases to the control list; i.e., these can be seen as individuals with the genomic risk to have CAD but no symptoms/diagnosis yet. We assumed that 10% of cases would be incorrectly labelled as controls, therefore if the population RCC is 0.05, roughly 0.005 of the samples in the control group would actually be incorrectly labelled cases. We assumed no controls would be incorrectly labelled as cases, therefore did not add any controls to the cases group.

**Fig 4:**
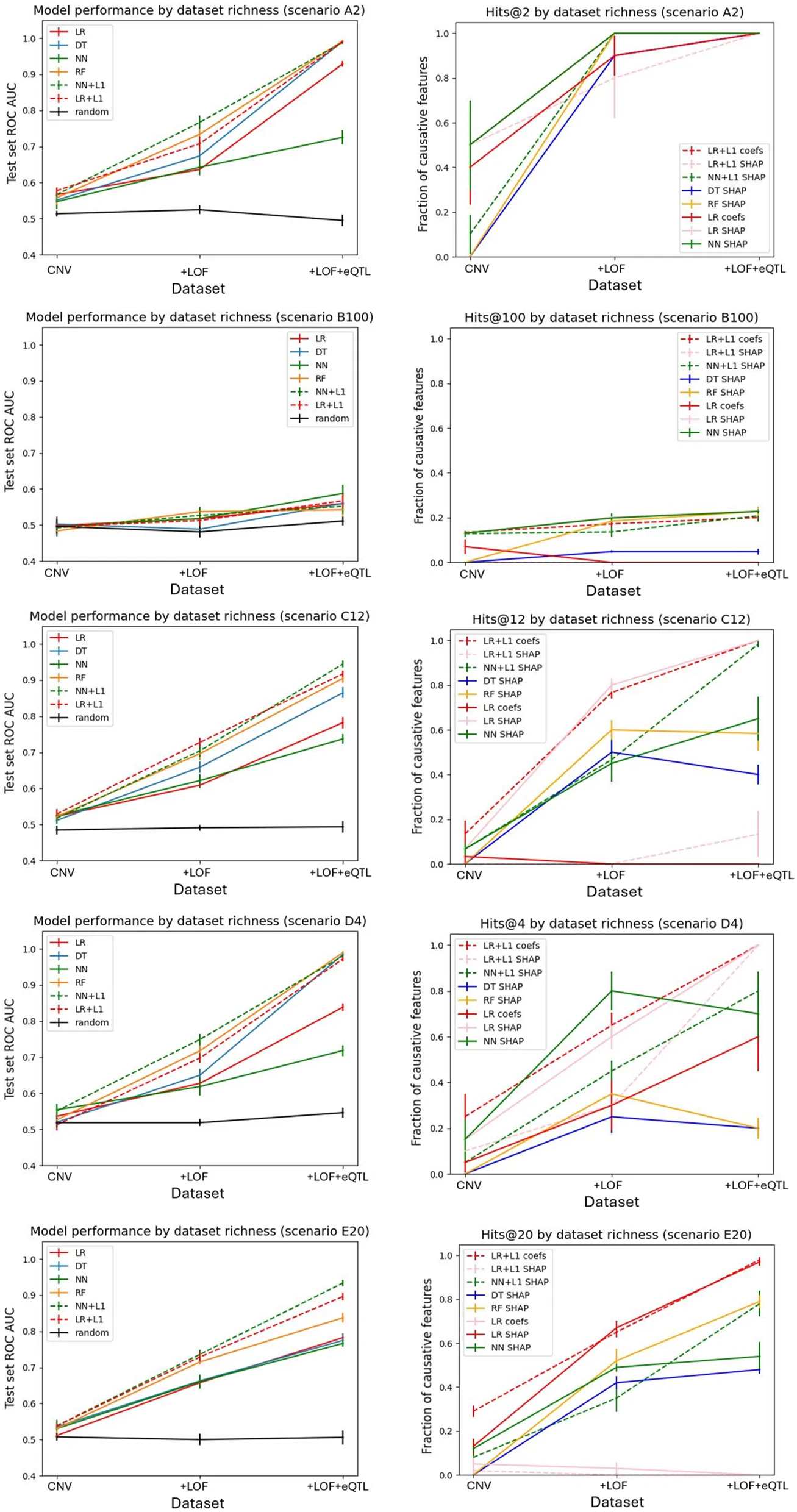
Model performance metrics by dataset richness for scenarios A2, B100, C12, D4, and E20. For each scenario, the test set ROC AUC and Hits@K are shown for CNV only, + LOF, and +LOF + eQTL datasets.

The results for the three different datasets, for each scenario, are shown in Figs 4 and S5. The + LOF + eQTL datasets are equivalent to the previous synthetic datasets except for the addition of label noise. Thus, it is not surprising that the classification performances achieved in these new scenarios are not very different from the previous ones. The results show how both the classification performance and the ability to identify CF decrease as the datasets become less rich; i.e., they lack information about specific types of variants. From these results we can see that the models tend to perform between 0.5 and 0.6 for the CNV-only dataset, which at least partially explains our lack in success when training models on the CNV-only UK Biobank datasets. As with the previous synthetic datasets (Figs 2 and S2), some ML approaches are able to identify the important contribution of some of the CFs in some genetic scenarios even when the classification performance is not very different from a random classification (S1 Table).

## Discussion

Here, we explored the potential for ML models to predict disease and detect causative genes using synthetic datasets representing a variety of disease scenarios. We found it was possible to achieve a good performance for most of these synthetic scenarios, and that identification of causative features did not require very high performance. Nonetheless, predicting cardiovascular diseases from real CNV datasets was much more challenging. Further investigations with synthetic datasets showed two possible reasons for the poor performance. These would mean that richer datasets containing information on the expression of functional genes would be necessary to obtain a high performance at disease prediction and causative gene detection. This agrees with the myriad of papers that have shown that phenotypes are associated with the expression of functional copies of the genes [77–79]. Thus, understanding the role of CNVs in the development of diseases will likely need to involve using LoF and expression data [26,57].

### Identification of causative features might be more accurate than classification

An important finding from this study was that it is possible for a model to be useful for feature detection even when the classification performance is not particularly high. This suggests that, for studies where feature detection is a key aim, it may be important to consider metrics such as Hits@K throughout all stages of the study, including any preliminary investigations, as it should not be assumed that a model with a low classification performance will not have any value in feature detection. As far as we are aware this has not previously been reported. We also observed that the LR model tended to be the most useful for feature detection, despite not performing better overall at classification. It is possible that this is due to LR models having a simple structure and being less prone to overfitting. We are not aware of other studies comparing feature detection relative to classification performance across multiple models, however LR models have previously been recognised as useful for feature selection [80–82]. However, for all other models we used SHAP values for interpretation, which were only calculated from a sample of the dataset. It is possible that, if this sample size was increased, we might see a capability for feature detection that is more in line with the LR coefficients for all other models, however increasing the sample size enough for this might be quite computationally expensive. One important consideration with regards to the difference between coefficients and SHAP values is that we used both values with LR models with and without L1 regularisation. Our results show that the SHAP values are not always at a clear disadvantage, and in some scenarios the advantage is clearly provided by the L1 regularisation. Taken together, these observations suggest that LR + L1 might be genuinely superior at identifying the causative features. However, further research would be necessary to confirm this.

### Gene-dosage models as a biologically-informed attempt to solve the dimensionality curse

ML has been used in several studies of Single Nucleotide Polymorphisms (SNPs) [83–88]. Nonetheless, the overall results have been far from a resounding success. Some ML approaches have slightly improved the performance of classical linear models when predicting some diseases or complex traits [85–88]; however, the additive models have proven themselves superior on other occasions [83,84,86–88]. The prediction performance is highly dependent on the number of samples and SNPs. Linear models seem to be superior with small samples [87], and a great number of samples is necessary for detecting non-linearity [85,87]. Our classification results agree with those previous observations: linear models are the best-performing methods when the number of samples is small, and at least 500 samples are necessary for accurately classifying non-linear scenarios. Previous studies have shown that the performance of ML approaches decreases when the number of SNPs (features) is too low or too high [83,85,86]. Given the high number of features, it is common to use only a subset of them; both the choice of SNPs [83] and the type of network sparsity [87] have been found to be relevant as well. Here we tried to overcome the dimensionality curse by focusing on the number of copies of protein coding genes; i.e., only around 20,000 features per sample, which could be reduced in number by removing invariable columns. However, the good classification performance of the algorithms when analysing synthetic data was not replicated in real data. The nature of previous ML applications to real CNV data make those studies unsuitable for comparison: studies either focused on classifying specific CNVs as pathogenic or benign [14,26,57,58], or they used unsupervised clustering to differentiate groups of cancer patients based on their normalised gene-specific CNV values [56]. In this later work, the authors found that the two groups of patient had different prognosis based on survival analysis, and this could be due to gene-dosage differences in particular genes [56]. However, this problem is very different to ours both in the targets and the features. First, they did not have target variables: all samples came from cancer patients and most patients died within one year (just 8 patients were alive after 500 days) [56]. Then, cancerous cells likely contain more CNVs than normal cells [89–91], in addition to multiple SNPs and other structural variants [92–94]. This would be quite a different problem from the ones we attempted to tackle: 1) predicting the risk of CAD, or 2) differentiating between individuals with BAV and healthy individuals or individuals with old-age valve disease. In our datasets, there were no substantial differences in the number of genes with deletions or duplications between cases or controls; we assume that the relevant factor is which gene has the gene-dosage alteration.

### Difficulty of modelling the complexity of real-world genetic scenarios

One possibility for the discrepancy in performance between synthetic and real data could be that our genomic scenarios are too simple. Unfortunately, we have not found other similar biologically-informed synthetic datasets readily available to use. Although we simulated scenarios involving up to 200 causative features, none of our scenarios required simultaneous CNVs in more than 3 of those features; i.e., they were not polygenic diseases (e.g., involving the contribution of more than 20 genes per sample) even if there were many possible causative genes. Scenarios A and D would have a similar inheritance pattern to many cases of Hypertrophic Cardiomyopathy [95,96]. Scenarios B and C resemble some cases of Long QT syndrome [97], non-syndromic deafness [98], Bardet-Biedl syndrome [99–101], hypogonadotropic hypogonadism [102,103], skeletal muscle myopathy [104], and albinism [105]. Scenario E is a combination of Scenario B plus a trigenic phenotype [106,107]. It could be seen as an oligogenic disease with epistasis; i.e., three variants are generally necessary, but two variants can be enough to cause the disease if they occur in specific pairs of genes. Thus, it is possible that the 3 diseases that we analysed might have many more causative genes than we included in our simulations, each of them only making a small contribution. For example, even if some cases of familial monogenic CAD have also been found, most cases of CAD are probably polygenic [108]. This mixture of case origins may result in both the lack of significant gene-dosage enrichments or depletions when analysing the whole genome, and the possible identification of significant differences if using prior knowledge to study a single gene. Moreover, the null effect of the data transformation approaches points towards genetic scenarios in which the causative values are not uniformly distributed; e.g., some genes will contribute to disease if their gene-dosage is altered, other genes only when deleted, and others only when duplicated. Thus, the causative combinations of concurrent changes would be more complex than we simulated; e.g., the disease would occur when enough of those causative features contributed (i.e., they had the relevant gene-dosage change) but few (if any) features would be essential for the development of the disease. Taken together these two factors would lead to a combinatorics explosion. A possible consequence would be that the vast majority of cases in the dataset would have a unique combination of concurrent gene-dosage alterations in the causative genes. In that case, we could only dream of using ML approaches with datasets much bigger than the ones we have now. Although this pessimistic view may seem sensible, it runs contrary to the evidence that some studies have identified causative gene-dosage alterations using statistical approaches [2,4,6,12,13]; e.g., our analyses of UK Biobank data showed that a few genes had significant gene dosage differences when tested individually hence targeted approached based on prior knowledge might succeed. Moreover, our analyses of simulated data suggest that some ML methods do not need to observe all combinations in order to be able to build a generalizable model; i.e., scenarios C120, E20 and E200 involve more than 1,000 possible causative combinations but only hundreds of samples seem to be necessary to achieve a classification performance clearly better than chance and to identify a substantial number of the causative features (see Figs 2 and S2). In the case of scenarios E20 and E200 it could be argued that 10 or 100 pairs, respectively, may be enough to include all causative features. However, 10,800 different combinations are possible in scenario C120. This demonstrates that the algorithms are able to generalise the contribution of causative features to unseen combinations. A final consideration is that using larger datasets should help improve the classification performance, especially if combinations of causative features are uncommon (Figs 2 and S2–S4); however, computational constraints may dictate how large the datasets can be.

### Effect of data richness on the classification performance

We found that the lack of success in attempting to predict cardiovascular diseases based on gene-dosage data could be attributed to data incompleteness; i.e., the label is influenced by additional genomic variants. Training models on synthetic datasets of CNV data with labels calculated from a richer dataset resulted in test set ROC AUCs from 0.5-0.6 (see Figs 4 and S5). This is still slightly higher than the value of 0.5 we obtained on the ground truth datasets. It is possible that the genetic scenarios behind the diseases we were trying to predict are quite complicated (e.g., some variants may have incomplete penetrance) or have a high number of CFs (e.g., polygenic or quasi-omnigenic diseases). Another possibility is that the label noise value of 0.1 used in these synthetic simulations is an underestimation. Congenital diseases such as BAV are the only ones present from birth; the rest of genetic diseases will appear latter. This means that many individuals will be initially considered controls, and they will become cases later even if their gene-dosages are the same all their life. This means that our control set may have included a proportion of individuals who will develop CAD in the future. Our results here suggest that training models using just gene-dosage data is likely to be unsuccessful in many diseases, ideally functionality and regulation of expression data would also be used. However, if only CNV and WES data are available (i.e., getting a good estimate of the number of functional copies of each gene), our results suggest that depending on the scenario it may still be possible to predict disease and detect causative features to a reasonable standard.

### Limitations and further directions

A limitation of this work is that the estimated frequency values for functionality errors and regulation in gene expression may not be totally accurate. Ideally an analysis of how results are affected when these values are varied should be carried out. Additionally, we assumed that all genes were equally likely to have a CNV, be non-functional, or have their expression affected. In reality, some genes will tolerate those changes better than others: 1) some variants may be lethal and hence be never observed in a biological dataset; 2) some genes may be very resilient to changes as far as there is at least one functional copy; 3) some variants may be more or less abundant because of population stratification, and, 4) since probands with syndromic diseases were removed from the biological dataset, dosage changes affecting genes strongly linked to those diseases might be unobserved (e.g., probands with trisomy 21 were filtered out from the dataset hence many of the duplications affecting genes in chromosome 21 were likely not observed in the remaining data). Moreover, we inflated the variant-to-feature ratio to avoid a large proportion of control samples with no variants at all. As a consequence, there were some differences between the synthetic and biological datasets with regard to the distribution of changes (e.g., see S6 Fig), which may have affected the performance of the ML algorithms. One such difference is that there were very few (if any) invariable features in our synthetic dataset, but we could dramatically reduce the size of our biological datasets because many features were invariable. Thus, it is possible that the noise in our synthetic datasets was relatively uniformly spread across more features than in the biological datasets. Alternatively, the use of a similar number of variants but fewer features can be interpreted as if we concentrated all the variants in fewer features and we made 19,000 features invariable. It would be important to explore the effect of the data structure (number of features, number of variants and distribution of variants) in the performance of the different ML methods.

There are many other lines of potential further investigation leading on from this work. Firstly, the algorithms tested here should be used to try to predict diseases with a richer real dataset containing functionality and expression data. There are also further areas to explore with the synthetic datasets. We have used a small number of potential disease scenarios and ML algorithms here; so, it would be interesting to extend this work to a greater variety of scenarios –especially, scenarios in which different features have different contributions- and to use other algorithms (e.g., models that can make use of spatial information). Furthermore, the influence of the environment (either as a risk factor or compensating the genetic risk) or confounders (e.g., sex) could be included in the simulations; e.g., by drawing a value from a Beta distribution and changing the label if a certain condition is met. Another interesting area to explore is investigating the correlation between feature rankings (from SHAP values/model coefficients) from various models and whether feature detection can be further improved by combining these rankings. Such further investigations could be supported by the open-source simulation framework we have developed here, which produces realistic synthetic datasets of varying richness with adjustable parameters for label noise and variant frequencies.

## Methods

### Dataset generation

Synthetic datasets are created by randomly creating samples until there are enough cases and controls (see Fig 1). The first step to creating a sample is to generate Markov chains of length equal to the number of features (NF) with transition matrix and steady state vector determined by the CNV frequency. The steady state vector is represented as ${[\pi }_{\text{0}},\pi_{\text{1}}]$, where $\pi_{\text{0}}\;$ is the probability of a state being 0 (normal gene-dosage) and $\pi_{\text{1}}$ is the probability of a state being 1 (CNV); i.e., $\pi_{\text{1}}$=the CNV frequency and $\pi_{\text{0}}$=1- CNV frequency. We also want the average number of consecutive 1s to be 2.1, based on an estimate of the number of consecutive genes affected by CNVs (estimation based in the analysis of CNVs in chromosome 1 from a sample of 10,000 probands from UK Biobank). From the equation:


$$\begin{array}{c} \text{E}\left[l_{ij}\right]=\;\frac{\text{1}}{\text{1}-p_{ij}} \end{array}$$

where E[*l*_*ij*_] is the expected length of consecutive *i* and *j* states (*i* and *j* equal to 1 in this case), the 1–1 entry $\left(p_{\text{1}\text{1}}\right)$ of the transition matrix can be calculated to be 0.527. Therefore, the 1–0 entry $\left(p_{\text{1}\text{0}}\right)$ is 1-0.527 = 0.473. We used a reversible Markov chain so that the samples were not dependant on the direction of generation; i.e., vectors generated in the forward and backwards directions would be equivalent. In order for a Markov chain to be reversible it must satisfy:


$$\begin{array}{c} \pi_{i}P_{ij}=\pi_{j}P_{ji};\;all\;i,j \end{array}$$

Therefore we can calculate $p_{\text{0}\text{1}}$ as:


$$\begin{array}{c} p_{\text{0}\text{1}}=\frac{p_{\text{1}\text{0}}*\pi_{\text{1}}}{\pi_{\text{0}}} \end{array}$$

And $p_{\text{0}\text{0}}$ will be 1-$p_{\text{0}\text{1}}$. In the first set of simulations, we used 0.05 as the CNV frequency, this results in $p_{\text{0}\text{1}}=\text{0}.\text{0}\text{2}\text{4}\text{9}$ and $p_{\text{0}\text{0}}=\text{0}.\text{9}\text{7}\text{5}$. In the second set of simulations, we estimate CNV frequency to be 0.0003 for coding genes from the prevalence of CNVs in the UK Biobank data. As we use a NF of 1000 rather than 20,000 in our simulations, we increase the CNV frequency by 20 to maintain the same difficulty level for modelling. Therefore, our CNV frequency is 0.006, which gives us $p_{\text{0}\text{1}}=\text{0}.\text{0}\text{0}\text{2}\text{8}\text{5}$ and $p_{\text{0}\text{0}}=\text{0}.\text{9}\text{9}\text{7}$. As a result, the number of CNVs in our synthetic data was similar to that of real-world biological data; however, the distribution of changes was clustered in 1,000 features in the synthetic data and spread in a larger number of features in the real-world data (a proportion of genes are intolerant to variation hence the number of features with variation is smaller than 20,000).

Next, the chain is converted from a string of 0s and 1s to a string of numbers from 1-4, representing copy numbers. First, all 0s are replaced by 2s, as this is the ‘normal’ state. Then, some groups of 1s are replaced with 3s or 4s to represent duplications, whilst some are left as 1 to represent deletion. The proportion of CNVs that are deletions, duplications, or double duplications are 0.404, 0.582, and 0.014 respectively, again this is estimated from the UK Biobank data. To decide what type of CNV a string of 1s will be converted to, a random number is generated using Pythons Random module and the interval this number falls into determines the CNV type.

For the model performance vs NS analysis, a label is now assigned to the sample. For the model performance vs dataset richness scenario, we next randomly add expression and functionality information. First, for each feature in the sample a random number is generated to determine whether to subtract 1 for a loss-of-function variant. The probability of this is estimated to be 0.0008, which is scaled up to 0.016 in line with the NF reduction. Next, another random number is generated for each feature to represent genes affected by a variant that increases (add 1) or decreases (subtract 1) its expression. The probabilities for these are estimated as 0.0002 and 0.0008 respectively, scaled up to 0.004 and 0.016. These additions and subtractions mean that the number of expressed functional copies for some features in the dataset might be below 1 or above 4, though this would be a rare phenomenon due to the low prevalence of all the variants. Finally, the label is calculated from this last dataset. Labels only depend on the values of the causative features; the rest of features in the dataset are irrelevant when labelling the samples.

Case or control labels are assigned for each sample depending on the disease scenario and the values of the CFs. These were spaced evenly throughout the sample.

### Model development

Three models used sci-kit-learn functions [109]; LogisticRegression, Tree, and RandomForestClassifier. For all these models, the sci-kit learn function GridSearchCV was used for hyperparameter optimisation: regularisation strength was optimised for the Logistic regression and ccp_alpha was optimised for the decision tree and random forest models. The Logistic regression model was trained both with default values for the penalty and solver and with L1 regularisation and liblinear solver (as default solver lbfgs did not support L1 penalty). Default values were used for all other hyper-parameters apart from class weight which was always set to balance out the class size inequality. The NN was constructed using TensorFlow [110], it had one hidden layer with 500 hidden units. To train the NN the RMSprop optimizer was used with a base learning rate of 0.01. The NN model was trained with and without L1 kernel regularization with regularization factor = 0.001.

### Calculating metric values and displaying results

The ROC AUC was calculated for each model training attempt using the scikit-learn function roc_auc_score. For Hits@K and last rank, SHAP values were calculated for each model using the SHAP framework in python, and features coefficients were also extracted for the logistic regression model. The Hits@K and last rank values could then be found by sorting the SHAP values using the in-built sort() python function, and matching SHAP values with feature indices using the numpy.where() function to determine which features were CFs. Graphs were then produced for each metric using the matplotlib python library.

### Preparation of UK Biobank data

CNVs were mapped to a list of all genes with chromosome, start and stop coordinates (GRCh37) as given by ensembl v111 [111]. If any of part of the CNV overlapped the gene then it is considered a hit and the copy number of that gene is deduced from the CNV call. Where genes have no CNV in an individual the copy number is assumed to be ‘2’. Originally, each sample had a copy number for every gene. To reduce the number of features for modelling, we filtered out genes that were non-coding.

### Data transformation and dimensionality reduction

We used two different data transformations on the real-world data: standardisation and value replacement to create linear separation.

Standardisation consists of the scaling of values by subtracting the mean and then dividing by the standard deviation. This is a common method used when training ML models as it balances the impact of features which can improve model performance.

Any scenario in which a causative feature contributes to the case label because of an abnormal gene dosage, that is different to 2, is not linearly separable. It is not possible to find a single line or hyperplane that separates cases and controls; it would need at least two. By replacing the normal gene dosage value of 2 with −1, we can linearly separate them: controls would have a negative value while cases would have a positive value. Scenarios A-C and E would be in this situation. Conversely, any scenario in which only one type of gene-dosage variation (either gene deletion or gene duplication) is responsible for making a feature causative is already linearly separable: it is possible to find a line between two values that separates cases and controls. In those scenarios, the replacement of 2s with -1s will keep the linear separability if the causative variation is the gene duplication, but it will remove it if the gene deletion is responsible. Scenario D would be in this situation. Given that disease cases in real-world data may be caused by different genetic scenarios (e.g., some cases having monogenic inheritance, whereas other cases being polygenic) it is likely that we did not achieve complete linear separability, but replaced some linearly separable features by others.

The only dimensionality reduction technique that we used was the removal of invariable features from the datasets. This hardly had any effect in the synthetic datasets but reduced the size of the real-world datasets. For example, out of 20343 coding genes within the UK Biobank dataset, we removed around 63% of the genes because of lack of gene-dosage variation: we retained 7264 genes in the CAD vs control dataset and 7696 genes in the BAV vs AV65 dataset.

### Functional and phenotypical analysis of top contributors

Functional enrichment analyses were performed using g:Profiler (https://biit.cs.ut.ee/gprofiler/gost) [72] and DAVID (https://davidbioinformatics.nih.gov/) [67]. No electronic GO annotations were used in g:Profiler. Only disease annotations were used when running DAVID. The rest of parameters were set at default values.

Association of top-contributing genes with cardiovascular effects was searched in OMIM (https://omim.org/) [112]. DisGENET (https://disgenet.com/) [113] was used to identify genes associated with bicuspid aortic valve disease (UMLS CUIs: C0149630, C4476977, C4476978, C4476982), age-related aortic valve disease (UMLS CUIs: C0003504, C0003507, C0428791) and CAD (UMLS CUIs: C0010073, C0242231, C0948089, C1867743, C1956346). The GWAS Catalog (https://www.ebi.ac.uk/gwas/) [114] was also used to identify genes associated with bicuspid aortic valve disease (EFO ID: HP_0001647), age-related aortic valve disease (EFO ID: EFO_0009531) and CAD (EFO ID: EFO_0001645).

## Supporting information

S1 Fig Diagram showing the steps involved in the ML modelling. A. The full dataset is randomly split in a 80:20 ratio into a training set and a test set. B. The training set is used to find the optimal hyperparameters through a 5-fold cross validation process. This means that the training set is divided in 5 equal size subsets: each of the subsets will be the validation set once, while the rest of subsets will be used for the hyperparameter tuning. C. The whole training set and the optimal hyperparameters are used to build the ML model. D. The performance of the ML model is assessed using the test set.
(TIF)

S2 Fig Model performance metrics by number of samples for scenarios A20, B10, C120, D40, and E200. For each scenario, the test set ROC AUC and hits@k are shown for dataset sizes from 50−10,000.
(TIF)

S3 Fig Additional model performance metrics by number of samples for scenarios A2, B100, C12, D4, and E20. For each scenario, the number of CFs found and the rank of the last CF found are shown for dataset sizes from 50−10,000.
(TIF)

S4 Fig Additional model performance metrics by number of samples for scenarios A20, B10, C120, D40, and E200. For each scenario, the number of CFs found and the rank of the last CF found are shown for dataset sizes from 50−10,000.
(TIF)

S5 Fig Model performance metrics by dataset richness for scenarios A20, B10, C120, D40, and E200. For each scenario, the test set ROC AUC and Hits@K are shown for CNV only, + LOF, and +LOF + eQTL datasets.
(TIF)

S6 Fig Distribution of CNVs across all genes for each UK Biobank cohort. The proportion of genes that are invariant or have at least one CNV (left) and the number of CNVs in non-invariant genes (right) for: **A)** CAD group, **B)** Control group, **C)** BAV group, and **D)** AV65 group.
(TIF)

S1 Table Expected Hits@K performance if selecting K features at random. (DOCX)

S1 File S2–S3 Tables. (XLSX)

S2 Table Number of genes that had a statistically significant (P < 0.05) difference in the number of CNVs across the Control and CAD populations and across the BAV and 65AV populations. (DOCX)

S2 File S5–S10 Tables. (XLSX)
