# Supplementary material for: Machine Learning approaches for the detection of disease-causing variants in whole-genome data need to address the expression of functional genes
Source: PLoS One. 2026 Aug 28;21(8):e0355557. doi: 10.1371/journal.pone.0355557 (PMC13524293; doi:10.1371/journal.pone.0355557)
Supplement: Supplementary file 1 [file pone.0355557.s007.docx]

**S1 Table.** Expected Hits@K performance if selecting K features at random.

| **Number of causative features** | **Number of randomly selected features** | **Fraction of causative features identified (95% CI)^1^** | |
| --- | --- | --- | --- |
|  |  | **2.5% percentile** | **97.5% percentile** |
| 2 | 2 | 0.000 | 0.000 |
| 4 | 4 | 0.000 | 0.000 |
| 10 | 10 | 0.000 | 0.100 |
| 12 | 12 | 0.000 | 0.083 |
| 20 | 20 | 0.000 | 0.100 |
| 40 | 40 | 0.000 | 0.100 |
| 100 | 100 | 0.050 | 0.160 |
| 120 | 120 | 0.067 | 0.175 |
| 200 | 200 | 0.150 | 0.250 |

^1^ 95% CI obtained from 10^6^ random selection experiments.
