# Supplementary material for: Machine Learning approaches for the detection of disease-causing variants in whole-genome data need to address the expression of functional genes
Source: PLoS One. 2026 Aug 28;21(8):e0355557. doi: 10.1371/journal.pone.0355557 (PMC13524293; doi:10.1371/journal.pone.0355557)
Supplement: Supplementary file 2 [file pone.0355557.s009.docx]

**S4 Table:** Number of genes that had a statistically significant (P<0.05) difference in the number of CNVs across the Control and CAD populations and across the BAV and 65AV populations.

|  | Control vs. CAD | BAV vs. 65AV |
| --- | --- | --- |
| Number of statistically significant genes before any corrections | 19 | 164 |
| Number of statistically significant genes after Bonferroni correction | 0 | 0 |
| Number of statistically significant genes after Benjamini-Hochberg Procedure* | 0 | 0 |

* Using a FDR level of 0.05
